# Method of Surgical Diagnosis Erichsen

**Published:** 1874-05

**Authors:** 


Methods of Surgical Diagnosis.—The eminent Mr. Erichsen, in a recent lecture reported in the London *Medical Times and Gazette*, says:

There are three methods that you may employ. The first and simplest method, and happily in surgery we have very simple methods of diagnosis, is by finding one pathognomonic sign. By "pathognomonic" is meant a thing which of itself indicates the nature of a condition. For instance, a person complains of dimness of vision. You look into his eyes, and you see an opacity of the lens. That of itself determines at once the nature of his disease, cataract. You need not ask him a single question or go a step further. Again, a person complains of trouble about the bladder. You introduce a sound, and you feel a calculus and hear it struck. Thus at once a single sign, and that sign a pathognomonic one, is determinative in itself and by itself, not only of the existence of a malady. You determine by that single sign, not only the existence, but the very nature of the malady that exists. Well, in surgery alway seek for the pathognomonic sign, and endeavor to determine, if you possibly can, at once and by a single sign, what the patient's lesion may be.

Now the second method in surgery consists in getting what may be termed a "pathognomonic group" of signs; that is to say, a set of signs which singly and individually are not indicative of any one given disease or injury, but which, taken collectively as a group, indicate incontestably the nature of some given injury or disease. Take, for instance, the case to which I have already alluded, of an elderly person being tripped up upon the floor and being unable to rise. You look at the limb and find that it is somewhat shortened, that it is everted, that the patient is unable to raise it off the ground, that he complains of considerable pain, and that you feel crepitus about the region of the hip. Now any one of these signs, shortening of the limb, eversion of it, inability to move it, and crepitus, any one of these signs is common to a variety of different injuries and diseases of the lower extremity; but the group, taking them collectively, is indicative of only one single condition, and that condition is fracture of the neck of the femur. Hence, although the indiviual signs may be untrue in themselves, so far as the determination of any given injury is concerned, they are absolutely true, and incontestibly so, when grouped together, in determining the nature of the particular injury. That is the second method, then, of effecting a surgical diagnosis, by getting a pathognomonic group of signs or symptoms, for it will do for either.

The third method is a very important one, and it is the method that was greatly employed in the French school of surgery, and the employment of which undoubtedly led to the high position that it occupied, and does occupy, as a diagnostic school. It is what may be termed the negative method, or what is termed by French surgeons the "method by exclusion." By this method you first of all ascertain what a thing is not, and then by excluding everything that is not, you arrive at last at what it is. It seems a roundabout way of arriving at the truth, but in point of practice it is an exceedingly simple way. A patient comes to you with a tumor in the scrotum. You are in doubt as to what it is. You examine it first of all by transmitted light. You find that it is not translucent; therefore it is not a hydrocele. You examine the upper part; you find there is no impulse on coughing, and that the cord is not covered; therefore it is not a hernia. You find that the cord itself is not enlarged, is not tortuous, and vermiform in its feel; therefore it is not a varicocele. Having removed hydrocele, hernia, varicocele from any possible tumor of the scrotum, what have you left? Why two conditions, hæmatocele and sarcocele. You find that it has not followed a blow, that it is not globular and uniform, that the scrotum is not discolored; therefore it is not a hæmatocele, *ergo*, it must be the last of these conditions, and that is a tumor of the testicle; a sarcocele. In that way, by determining what a thing is not, you speedily arrive at what it is; and this determination, in the hand and in the mind of a practiced surgeon is so rapid as to be almost instantaneous. The whole process is gone through in his mind with such rapidity that he lays his hand upon the part he feels for everything, and he finds that four out of five conditions are absent; and his diagnosis is made instantaneously, although it is made by that process of negation or exclusion, and though the steps that lead to it are apparently complicated.

